# Effects of Inhaled Corticosteroids on Lung Function in Children With Post-infectious Bronchiolitis Obliterans in Remission

**DOI:** 10.3389/fped.2022.827508

**Published:** 2022-05-10

**Authors:** Haoqi Zheng, Xiuhua Yu, Yuquan Chen, Wenying Lin, Li Liu

**Affiliations:** ^1^Department of Pediatrics, The First Hospital of Jilin University, Changchun, China; ^2^Institute of Medical Information/Library, Chinese Academy of Medical Sciences and Peking Union Medical College, Beijing, China

**Keywords:** corticosteroids, post-infectious bronchiolitis obliterans, budesonide, children, lung function

## Abstract

**Background:**

Post-infectious bronchiolitis obliterans (PIBO) is a rare and irreversible chronic obstructive pulmonary disease with no specific treatment, especially for patients with PIBO in remission. In this study, we evaluated the effects of continuous inhaled corticosteroids (ICSs) and intermittent ICSs on lung function in the remission of PIBO.

**Methods:**

This was a retrospective study, and all the subjects we included were divided into continuous ICS group and intermittent ICS group according to treatment regimens. Patients in continuous ICS group received continuous ICSs (2 times a day), and patients in intermittent ICS group received intermittent ICSs (inhaled corticosteroids after acute respiratory tract infection or wheezing). Different lung function tests were performed at different ages. Tidal breathing lung function tests were performed in patients with PIBO aged ≤ 5 years, and the lung ventilation function test and the bronchial dilation test were performed in patients with PIBO aged more than 5 years. Lung function was assessed at the beginning of follow-up and at the end of follow-up (1 year of ICSs).

**Results:**

After 1 year of ICSs, patients aged more than 5 years, forced vital capacity (FVC), and forced expiratory volume in 1 s (FEV_1_) were significantly higher than at the beginning of follow-up. After 1 year of ICSs, the difference in V_T_/Kg, TPTEF/TE, and VPEF/VE between the end and the beginning of follow-up in continuous ICS group shows an upward trend. But those showed a downward trend in intermittent ICS group. FVC, FEV_1_, and maximal mid-expiratory flow velocity 25–75% (MMEF_25–75%_) of continuous ICS group were significantly higher than at the beginning of follow-up. The difference in FEV_1_ and MMEF_25–75%_ between the end of follow-up and the beginning of follow-up in continuous ICS group was significantly higher than that in intermittent ICS group. A total of 52.94% of patients with PIBO aged more than 5 years were positive for bronchial dilation tests.

**Conclusion:**

Inhaled corticosteroids can effectively improve lung function and relieve airway obstruction in patients aged more than 5 years in PIBO remission, especially continuous ICSs. Patients with PIBO may have reversible airflow limitations.

## Introduction

Post-infectious bronchiolitis obliterans (PIBO) is an irreversible chronic obstructive lung disease commonly seen in children with severe lower respiratory tract infections secondary to adenoviruses (ADV), *Mycoplasma pneumoniae* (MP), and other pathogens. At present, the pathogenesis of PIBO has not been explored, but most research evidence suggested that lung injury of PIBO was caused by inflammation and immunity. After being injured, the bronchioles may gradually form fibrosis, forming irreversible narrowing and leading to partial or complete occlusion of the airway lumen (1).

Currently, there is no clear and appropriate therapeutic regimen, which mainly referred to the treatment experience of bronchiolitis obliterans after bone marrow transplantation and stem cell transplantation. In addition, symptomatic support treatment is the basis, such as respiratory support and nutritional support (2). At present, the symptoms, signs, and lung function of patients with PIBO were improved in most treatment centers after corticosteroids, macrolides, and other basic drugs and symptomatic supportive treatment (3–5). A study found that lung function can be improved after corticosteroid therapy, which would gradually deteriorate again without corticosteroid maintenance therapy (6). Because long-term methylprednisolone pulse or oral corticosteroids can cause complications, such as osteoporosis, hypertension, and hyperglycemia. Inhaled corticosteroids (ICSs) can avoid systemic corticosteroid adverse reactions and reduce airway hyperreactivity, which can be used as a treatment for patients with PIBO in remission. Budesonide is a commonly used inhalant for patients with PIBO.

Budesonide can affect the metabolism of arachidonic acid, reduce the synthesis and release of leukotriene and prostaglandin, inhibit the synthesis and activity of eosinophils, and improve airway hyperreactivity (7). Zhang et al. (8) found that after 12 months of inhaled budesonide combined with terbutaline, the clinical symptoms and lung function of PIBO were improved. In addition, Chen et al. (9) showed that budesonide, montelukast, and azithromycin can improve lung function in the acute stage of PIBO, which has positive therapeutic significance.

However, it is not known whether ICSs can prevent or reverse the decline in lung function during PIBO remission. In this study, the aim was to evaluate the efficacy of inhaled budesonide in PIBO remission through changes in lung function and to compare the effects of continuous ICSs and intermittent ICSs in lung function, so as to guide the therapeutic regimen of PIBO remission.

## Materials and Methods

### Study Subjects

This study retrospectively analyzed the clinical data and lung function results of 34 patients with PIBO who received treatment and follow-up at The First Hospital of Jilin University from 2007 to 2021. This study was approved by the ethics committees of our research institutions (no. 2021-714). All participant patients and their parents or legal guardians agreed to the research inclusion.

### Diagnostic Criteria and the Setting of the Research Starting Point

The diagnosis of PIBO was made according to the following diagnostic criteria (10, 11): (1) persistent or repeated coughing, wheezing, tachypnea, and exercise intolerance after severe lower respiratory tract infection, persisting for at least 6 weeks; (2) widespread wheezing and wet rales can be heard in both lungs; (3) mosaic perfusion signs, bronchiectasis, or bronchial wall thickening on chest high-resolution computed tomography (HRCT); (4) obstructive ventilation disorder on lung function; and (5) exclude other diseases that can cause wheezing, such as severe asthma, cystic fibrosis, immunodeficiencies, and bronchopulmonary dysplasia.

Definition of PIBO in remission: At present, there are no corresponding diagnostic criteria for PIBO. This study referred to the diagnosis criteria of bronchial asthma in remission stage (12) and combined with the clinical manifestations and lung function of PIBO to diagnose the remission stage of PIBO. It refers to the relief of cough and wheeze after treatment, occasional (or not accompanied by) cough and wheeze, no progressive exacerbation of lung function and HRCT, and the maintenance of at least 3 months.

**FIGURE 1:**
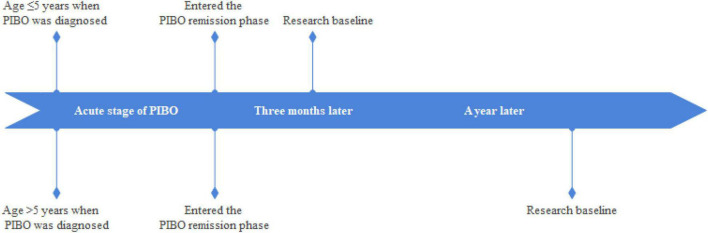
Flow chart of the setting of the research baseline.

The setting of the research baseline was as follows: (1) patients with PIBO aged ≤ 5 years to pass through the acute phase of the disease and complete the acute phase treatment regimen, and continuous ICSs for 3 months. (2) patients with PIBO aged > 5 years to pass through the acute phase of the disease and complete the acute phase of the treatment regimen, and continuous ICSs for 1 year (Figure 1).

### Inclusion and Exclusion Criteria

Inclusion criteria were as follows: (1) The age of the patients is ≤14 years; (2) meeting the diagnostic criteria of PIBO; and (3) meeting the conditions of the research baseline.

Exclusion criteria were as follows: (1) bone marrow transplant or organ transplant patients; (2) patients with connective tissue disease; (3) systemic corticosteroids were administered within 3 months prior to the study; and (4) patients with incomplete clinical data.

### Treatment and Grouping of Patients With Post-infectious Bronchiolitis Obliterans

All enrolled patients received oral low-dose corticosteroids (prednisone, 1–2 mg/kg/day, gradually reduced, and stopped in 3–6 months), oral azithromycin (5 mg/kg/day, 3 days per week, and 3–6 months), and inhaled budesonide (0.5 mg, two times a day). During the course of the disease, if there was an exacerbation of acute respiratory infection, medication regimens or corticosteroid doses need to be adjusted. After the patient entered remission, inhaled budesonide maintenance therapy was performed (two times a day). The changes in lung function were observed after 1-year follow-up.

**FIGURE 2:**
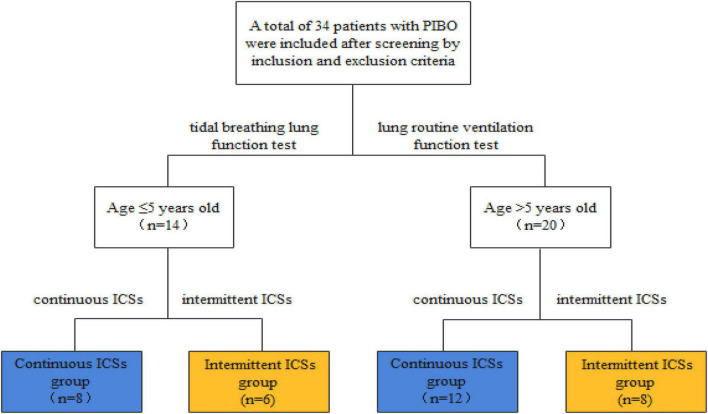
Study design.

The selected patients were divided into continuous ICS group (*n* = 20) and intermittent ICS group (*n* = 14) according to the different treatment regimens. Patients in continuous ICS group were treated with continuous ICSs (0.5 mg/time, two times a day) during remission, and patients in intermittent ICS group were treated with ICSs (0.5 mg/time, up to 3 times a day) after an acute respiratory infection or when cough and wheezing recurred, with intermittent treatment according to the symptoms. The interval between different courses of treatment was ≥3 months, and each course was ≤7 days (Figure 2).

### Lung Function

Patients ≤5 years cannot cooperate with forced breathing and generally conduct the tidal breathing lung function test (13, 14). The main indicators of lung function reflecting obstructive ventilatory disorder included: time to peak tidal expiratory flow as a proportion of expiratory time (TPTEF/TE), volume to peak expiratory flow as a proportion of exhaled volume (VPEF/VE), and the tidal volume per kilogram of body weight (V_T_/Kg). TPTEF/TE and VPEF/VE are the important indicators that reflect obstructive ventilatory disorder with a normal range of 28–55%. According to the different degrees of obstruction, they are divided into mild obstruction (28–23%), moderate obstruction (22–15%), and severe obstruction (<15%; 15). V_T_/Kg is an indicator of lung volume, which standard value for children is 6–10 ml/kg, lower than which is often indicative of restrictive pulmonary disease.

Patients aged more than 5 years conduct the lung ventilation function test and the bronchial dilation test. The main indicators of lung function reflecting obstructive ventilatory disorder included (expressed as a percentage of the estimated value): forced vital capacity (FVC), forced expiratory volume in 1 s (FEV_1_), FEV_1_/FVC, maximal mid-expiratory flow velocity 25–75% (MMEF_25–75%_).

Lung function is measured by trained physicians using Jaeger Master Screen Paed (Jaeger Company, Wurzburg, Germany) according to the Standards of the American Thoracic Society/European Respiratory Society. The lung function of patients with PIBO in remission was followed up for 1 year by the same doctor using the same equipment to ensure the accuracy and repeatability of the results.

### Statistical Analyses

The Shapiro–Wilk methods were used to test for normality. Normally distributed quantitative data are expressed as the mean ± standard deviation, and non-normally distributed data are described by median and interquartile ranges. The comparison between the endpoint and the baseline was performed by the paired *t*-test, and the independent sample *t*-test was used to compare the differences in lung function between two groups. Categorical data are described as numbers and percentages (%). Categorical variables were compared using the chi-square or Fisher’s exact test. A *p*-value < 0.05 was considered to be statistically significant. The statistical analyses were performed using Statistical Package for the Social Sciences version 20.0 software.

## Results

### Clinical Characteristics of Patients

Table 1 shows the clinical characteristics of 34 patients with PIBO. The median age was 22.41 months and ages ranged from 9 to 86 months. In addition, 67.6% of the participants were male. The pathogenic factors of all patients with PIBO included were severe pneumonia, 14 patients (41.2%) were infected with MP, 12 (35.3%) with adenovirus (ADV), 1 (3%) with measles virus infection, 1 (3%) with parainfluenza virus infection, and no specific pathogen was detected in 6 patients (17.5%). None of the patients had a family history of asthma. All patients had persistent or recurrent cough and wheezing after severe pneumonia, among which 11 (32.4%) had dyspnea and 13 (38.2%) had decreased exercise intolerance. All patients had mosaic perfusion signs on HRCT, and 5 (14.7%) of them had bronchial wall thickening. All the patients had moderate to severe obstructive ventilatory dysfunction. At the beginning of follow-up, all patients had occasional (or no) cough and wheezing, 2 patients (15.3%) still had decreased exercise intolerance, and the others had improved exercise intolerance. At the beginning of follow-up, TPTEF/TE and VPEF/VE were statistically different between continuous ICS group and intermittent ICS group (*p* < 0.05; Table 2). And there was no significant difference in lung function between continuous ICS group and intermittent ICS group on lung ventilation function (*P* >0.05; Table 3).

**TABLE 1:** Comparison of clinical data between continuous ICS group and intermittent ICS group at diagnosis of PIBO.

	Continuous ICS group (*n* = 20)	Intermittent ICS group (*n* = 14)	*p*-value
Gender, (M/F)	12/8	11/3	0.295
Age (months)	23.90 ± 17.77	20.28 ± 9.01	0.490
Symptom, *n* (%) ^a^			0.882
Cough	20 (100)	14 (100)	
Wheezing	20 (100)	14 (100)	
Tachypnea	8 (40)	3 (21.43)	
Decreased exercise intolerance	9 (45)	5 (35.71)	
Infectious pathogens, *n* (%)			0.904
ADV	7 (35)	5 (35.71)	
MP	9 (45)	5 (35.71)	
Others	4 (20)	4 (28.57)	

*^a^ is positive rate (each patient may have multiple clinical manifestations).*

**TABLE 2:** Comparison of clinical data between Continuous ICS group and intermittent ICS group at baseline (age ≤ 5 years old).

	Continuous ICS group (*n* = 8)	Intermittent ICS group (*n* = 6)	*p*-value
Gender, (M/F)	5/3	5/1	0.580
Age (months)	14.25 ± 5.57	17.33 ± 7.99	0.410
Symptom, *n* (%) ^a^			0.701
Cough	8 (100)	6 (100)	
Wheezing	8 (100)	6 (100)	
Tachypnea	1 (12.5)	0 (0)	
Decreased exercise intolerance	5 (62.5)	1 (16.67)	
Infectious pathogens, *n* (%)			0.326
ADV	2 (25)	3 (50)	
MP	3 (37.5)	3 (50)	
Others	3 (37.5)	0 (0)	
Lung function			
V_T_/Kg (mL/kg)	9.98 ± 1.75	9.83 ± 1.44	0.875
TPTEF/TE (%)	11.24 ± 2.93	18.93 ± 3.68	0.001
VPEF/VE (%)	18.16 ± 4.28	22.95 ± 3.20	0.041

*^a^ is positive rate (each patient may have multiple clinical manifestations).*

**TABLE 3:** Comparison of clinical data between continuous ICS group and intermittent ICS group at baseline (age > 5 years old).

	Continuous ICS group (*n* = 12)	Intermittent ICS group (*n* = 8)	*p*-value
Gender, (M/F)	7/5	6/2	0.642
Age (months)	30.33 ± 20.32	22.50 ± 9.59	0.325
Symptom, *n* (%) ^a^			0.848
Cough	12 (100)	8 (100)	
Wheezing	12 (100)	8 (100)	
Tachypnea	7 (58.33)	3 (75)	
Decreased exercise intolerance	4 (33.33)	4 (50)	
Infectious pathogens, *n* (%)			0.187
ADV	5 (41.67)	2 (25)	
MP	6 (50)	2 (25)	
Others	1 (8.33)	4 (50	
Lung function			
FVC (%)	55.72 ± 21.38	57.58 ± 20.16	0.848
FEV_1_ (%)	47.93 ± 22.26	47.75 ± 20.00	0.985
FEV_1_/FVC (%)	84.22 ± 20.10	81.83 ± 18.88	0.793
MMEF_25–75%_ (%)	29.06 ± 22.31	27.28 ± 16.74	0.850

*^a^ is positive rate (each patient may have multiple clinical manifestations).*

### Lung Function

Lung function tests were performed at the beginning of follow-up and after 1 year of treatment. In patients with PIBO ≤ 5 years, after 1 year of ICSs, TPTEF/TE and VPEF/VE in continuous ICS group and intermittent ICS group were not statistically significant compared with those at the baseline (*P* > 0.05; Table 4). This study further conducted a statistical comparison of the difference in lung function between the baseline and the end of the follow-up in the continuous ICS group and intermittent ICS group and found that V_T_/Kg, TPTEF/TE, and VPEF/VE in continuous ICS group showed an upward trend, and those in intermittent ICS group showed a downward trend. The improvement effect in continuous ICS group was slightly better than that in intermittent ICS group, but it did not reach statistical significance (*p* > 0.05; Table 5).

**TABLE 4:** Lung function before and after 1 year in patients with PIBO (age ≤ 5 years).

	All patients		Continuous ICS group		Intermittent ICS group	
Outcome	Baseline	Endpoint	Baseline	Endpoint	Baseline	Endpoint
VT/Kg (mL/kg)	9.91 ± 1.56	9.69 ± 1.16	9.98 ± 1.75	10.28 ± 0.81	9.83 ± 1.44	8.90 ± 1.12
TPTEF/TE (%)	14.53 ± 5.04	15.80 ± 4.88	11.23 ± 2.92	13.58 ± 4.89	18.93 ± 3.68	18.75 ± 3.17
VPEF/VE (%)	20.21 ± 4.45	20.57 ± 4.01	18.16 ± 4.27	19.18 ± 4.59	22.95 ± 3.20	22.43 ± 2.28

*represents p < 0.05.

**TABLE 5:** Difference in lung function before and after 1 year of treatment (age ≤ 5 years).

Outcome	Continuous ICS group	Intermittent ICS group	*p*-value
V_T_/Kg	0.31 ± 1.37	−0.93 ± 1.34	0.115
TPTEF/TE	2.35 ± 3.12	−0.18 ± 2.00	0.110
VPEF/VE	1.03 ± 2.35	−0.52 ± 1.83	0.209

In patients with PIBO more than 5 years, combined lung function after 1 year of ICSs, FVC (67.55 ± 19.01), and FEV_1_ (55.13 ± 23.43) were significantly higher than the baseline (*p* < 0.05; Table 6). After 1 year of ICSs, FVC (70.96 ± 21.92), FEV_1_/FVC (60.75 ± 24.95), and MMEF_25–75%_ (38.71 ± 25.61) of lung function in continuous ICS group were significantly higher than baseline (*p* < 0.05; Table 6). In intermittent ICS group, FEV_1_/FVC (71.69 ± 21.05) was significantly lower than the baseline (*p* = 0.037; Table 6), but there was no difference in the FVC, FEV_1_, and MMEF_25–75%_. This study further conducted a statistical comparison of the difference in lung function between the baseline and the end of follow-up in continuous ICS group and intermittent ICS group and showed that the improvement in FEV_1_ and MMEF_25–75%_ in continuous ICS group was significantly better than that in intermittent ICS group (*p* < 0.05; Table 7).

**TABLE 6:** Lung function before and after 1 year in patients with PIBO (age > 5 years).

	All patients		Continuous ICS group		Intermittent ICS group	
Outcome	Baseline	Endpoint	Baseline	Endpoint	Baseline	Endpoint
FVC (%)	56.46 ± 20.37	67.55 ± 19.01*	55.72 ± 21.38	70.96 ± 21.92*	57.58 ± 20.16	62.45 ± 13.29
FEV_1_ (%)	47.86 ± 20.84	55.13 ± 23.43*	47.93 ± 22.26	60.75 ± 24.95*	47.75 ± 20.00	46.71 ± 19.42
FEV_1_/FVC (%)	83.26 ± 19.15	79.46 ± 20.99	84.22 ± 20.10	84.65 ± 20.16	81.83 ± 18.88	71.69 ± 21.05*
MMEF_25–75%_ (%)	28.34 ± 19.80	32.92 ± 22.74	29.06 ± 22.31	38.71 ± 25.61*	27.28 ± 16.74	24.24 ± 15.15

*represents p < 0.05.

**TABLE 7:** Difference in lung function before and after 1 year of treatment (age > 5 years).

Outcome	Continuous ICS group	Intermittent ICS group	*p*-value
FVC	15.24 ± 13.23	4.88 ± 13.23	0.103
FEV1	12.82 ± 9.14	−1.04 ± 10.33	0.005
FEV1/FVC	0.43 ± 12.09	−10.14 ± 11.14	0.064
MMEF_25–75%_	9.65 ± 11.90	−3.04 ± 7.61	0.016

In addition, 9 (52.94%) of 17 patients who underwent bronchial dilation tests both times were found to be positive on both bronchodilation tests of lung function, FEV_1_ improvement rate ≥ 12% after inhaling bronchodilators. There was no statistical difference in the history of allergy and wheezing between the negative and positive bronchodilation test patients (*p* > 0.999, Table 8).

**TABLE 8:** Comparison of clinical data between patients with negative and positive bronchial dilation test.

	Positive (*n* = 9)	Negative (*n* = 8)	*p*-value
A history of allergy, *n* (%)	2 (22.22)	1 (12.50)	>0.999
A history of wheezing, *n* (%)	3 (33.33)	2 (25.00)	>0.999

### Safety Assessment

A total of 7 of 20 patients (35%) in continuous ICS group had respiratory tract infection (non-severe) during follow-up, whereas 5 of 14 patients (35.71%) in intermittent ICS group had respiratory tract infection (non-severe), with no statistical difference between the two groups. No complications or adverse events were reported in both groups.

## Discussion

This retrospective study that evaluates the effects of ICSs on lung function in patients with PIBO in remission found that ICSs can effectively improve lung function and relieve airway obstruction in patients with PIBO > 5 years of age in remission, especially continuous ICSs.

The intense and persistent inflammatory response is the key to the occurrence and development of PIBO. Studies have found that inflammatory factors, such as neutrophils and interleukin-8, were significantly increased in alveolar lavage fluid and sputum of patients with PIBO, which lasted for several years (16–18). Therefore, therapeutic interventions to prevent disease progression are to continuously suppress inflammatory responses throughout the course of the disease to prevent a persistent decline in lung function and reduce the severity of the disease. Corticosteroids can inhibit the release and aggregation of inflammatory factors, alleviate inflammatory reactions, and reduce airway hyperreactivity induced by respiratory tract infection and allergens, which is regarded as the basic and preferred medication for patients with PIBO (19). In most studies, corticosteroids have been shown to improve lung function and relieve clinical symptoms in patients with acute PIBO (9, 20), but the effect of corticosteroids on clinical manifestations and lung function of patients in remission of PIBO is still unclear. To combat the persistent inflammatory factors in the airway of patients with PIBO and the decline in lung function during remission, some treatment options need to be adopted. Therefore, this study evaluated the effects of ICSs on lung function in remission of PIBO and compared the effects of continuous and intermittent ICSs on lung function.

Lung function tests play an important role in diagnosis, severity assessment, and clinical efficacy evaluation (21). The lung function of patients with PIBO is usually characterized by severe fixed obstructive ventilatory disorder, with TPTEF/TE, VPEF/VE, FEV_1_, FEV_1_/FVC, and MMEF_25–75%_ decreased, and the volume of ventilation increased (22, 23). Patients with PIBO ≤ 5 years were tested for the tidal breathing lung function test. At the time of diagnosis, V_T_/Kg was within the normal range, whereas TPTEF/TE and VPEF/VE suggested moderate to severe obstructive ventilatory dysfunction. At the beginning of follow-up, the V_T_/Kg was still within the normal range, and 11 patients (78.57%) had moderate to severe obstructive ventilatory dysfunction. Thus, PIBO mainly causes airway obstruction and has no significant effect on lung volume. The findings of this study were consistent with the changes in the lung function of PIBO (10).

In patients with PIBO ≤ 5 years, after 1 year of ICSs, lung function of all patients showed that TPTEF/TE and VPEF/VE were not significantly increased compared with baseline. This study further compared the difference in lung function between the two groups and found continuous ICSs tended to improve lung function compared with intermittent ICSs, but it did not reach statistical significance. The reason why ICSs had no significant effect on lung function improvement in PIBO remission children ≤ 5 years old was analyzed. First, compared with conducting the lung ventilation function test, the tidal breathing lung function test is carried out under natural breathing conditions, which has its limitations. For some patients with rapid respiratory rate, TPTEF/TE and VPEF/VE will even rise, thus masking airway obstruction. The evaluation of the effect of ICS therapy on patients in remission of PIBO should not only consider lung function but also take into account the clinical manifestations and signs. The follow-up showed that the clinical symptoms and signs of 14 patients were better than before, and there was no progressive worsening of wheezing or decreased exercise intolerance during the follow-up period. It was considered that ICSs could play a positive role in the treatment of patients aged ≤ 5 years in the remission stage of PIBO. Second, it may be related to the sample size included in this study. By observing the lung function results of this study, it found that the difference in TPTEF/TE and VPEF/VE in continuous ICS group showed an increasing trend. While the difference in intermittent ICS group showed a decreasing trend, it suggested that airway obstruction was progressively aggravated in the intermittent ICS group. It was concluded that intermittent ICS group was not conducive to improve lung function and alleviate airway obstruction during PIBO remission, which needed to be further verified by large sample trials in the future.

Patients with PIBO aged more than 5 years were tested for lung ventilation function, in which FEV_1_, FEV_1_/FVC, and MMEF_25–75%_ are important indicators to reflect obstructive ventilatory disorder. In patients with PIBO aged more than 5 years, after 1 year of ICSs, lung function of all patients showed that FVC and FEV_1_ were significantly higher than the baseline, suggesting that ICSs can significantly relieve airway obstruction in patients with PIBO. Some studies have found that the improvement in lung function in patients with PIBO may be caused by the growth and development of lung tissue (24, 25), and this study did not conduct a controlled trial and cannot exclude the improvement in lung function caused by the growth and development of lung tissue itself. In particular, uneven growth of airway and lung parenchyma during lung tissue development in PIBO (25) and decline of FEV_1_/FVC were observed, which was consistent with the results of this study. At the end of follow-up, FVC and FEV_1_ in continuous ICS group were significantly increased compared with the baseline. FEV_1_/FVC in intermittent ICS group was significantly lower than the baseline and mainly showed a decrease in FEV_1_, suggesting progressive exacerbation of airway obstruction in the intermittent ICS group. Further comparison of the difference in lung function between the two groups found that FEV_1_ and MMEF_25–75%_ were significantly increased in the continuous ICS group. FEV_1_ and MMEF_25–75%_ are both flow indicators, and flow is mainly affected by the diameter of small and medium airways. The increase in these indicators indicated significant improvement in expiratory flow and relief of small airway obstructions. Therefore, this study suggested that continuous ICS can improve lung function and relieve airway obstruction in patients aged more than 5 years in remission of PIBO.

Previous studies have suggested that PIBO lesions present as fixed irreversible airway obstruction. However, this study found that 52.94% of patients with PIBO were positive in both bronchial dilation tests. Mattiello et al. found that 58.3% of patients with PIBO responded to bronchodilators, roughly consistent with the findings of this study (26). This study compared the history of allergy and wheezing between patients with negative and positive bronchodilation tests and found no statistical difference between the two groups. Previous studies have found that patients with PIBO with the positive bronchial dilation test are not significantly associated with a family history of asthma or allergy (26, 27). However, some studies have found that more than 50% of patients with PIBO have allergies, and this study suggested that PIBO can coexist with bronchial asthma (28, 29). It is difficult to say whether this was a concomitant allergy or a specific symptom of PIBO. At present, the mechanism of airway hyperreactivity in patients with PIBO is not clear. Some scholars found that patients with high airway reactivity were more likely to suffer from PIBO, and positive bronchodilation may occur in the later phase of the disease (27, 29); or whether it was related to repeated inflammatory stimulation and airway repair, promoting the formation of airway hyperresponsiveness. However, it should be noted that a study found that airway hyperresponsiveness in PIBO was different from that caused by eosinophilic or atopic airway inflammation in patients with asthma (30), and the specific pathogenesis is needed to be further explored in the future. Some studies have found that the simultaneous use of ICSs and bronchodilators can dilate bronchus and increase anti-inflammatory effects and reduce airway hyperresponsiveness (31). In recent years, bronchodilators have been used as a treatment for PIBO in many treatment centers.

Long-term use of corticosteroids will lead to a series of adverse reactions, such as serious infection, obesity, and osteoporosis. At present, the adverse effects of corticosteroids therapy in the acute phase of PIBO were rarely reported. In this study, 20 patients in remission of PIBO showed no obvious adverse reactions after 1 year of continuous ICSs. Moreover, compared with intravenous drip and oral administration, ICS acts locally in the airway with fewer adverse reactions and can be used for maintenance treatment of PIBO.

This study had some limitations. First of all, the study was retrospective and the sample size was relatively small, which may affect the persuasion of the research conclusions. Second, the follow-up was not long enough to cover the natural course of the disease, which may limit the assessment of long-term prognosis and adverse events.

Our study found that ICSs can effectively improve lung function and relieve airway obstruction in patients aged more than 5 years in PIBO remission, especially continuous ICSs. But the effect was not significant in patients with PIBO ≤ 5 years of age, which may be related to the small sample size, and large sample experiments are needed to further verify our conjecture. In addition, we found that patients with PIBO may have reversible airflow limitation, but the specific mechanism is still unclear and needs further research.

## Data Availability Statement

The original contributions presented in the study are included in the article/supplementary material, further inquiries can be directed to the corresponding author.

## Ethics Statement

The studies involving human participants were reviewed and approved by the Research Ethics Committees of First Hospital of Jilin University. Written informed consent to participate in this study was provided by the participants’ legal guardian/next of kin.

## Author Contributions

HZ undertook the follow-up of patients, data collection, and analysis and produced the manuscript. XY and WL were responsible for the follow-up of patients and data collection. YC undertook data analysis. LL was for the whole article and financial support as the corresponding author. All authors have read and approved the manuscript.

## Conflict of Interest

The authors declare that the research was conducted in the absence of any commercial or financial relationships that could be construed as a potential conflict of interest.

## Publisher’s Note

All claims expressed in this article are solely those of the authors and do not necessarily represent those of their affiliated organizations, or those of the publisher, the editors and the reviewers. Any product that may be evaluated in this article, or claim that may be made by its manufacturer, is not guaranteed or endorsed by the publisher.
